# Exploring Oat Husks as Aggregates in Limestone-Based Composites: Effects of Surface Treatments and Binder Selection on Mechanical Performance

**DOI:** 10.3390/ma17112588

**Published:** 2024-05-28

**Authors:** Alysson Larsen Bonifacio, Paul Archbold

**Affiliations:** Sustainable Infrastructure Research Group, Technological University of the Shannon, Midlands Midwest, N37HD68 Athlone, Ireland; paul.archbold@tus.ie

**Keywords:** oat husk, treatment, linseed, cement, mortar, composite, natural fibre

## Abstract

The viability of incorporating agricultural by-products, such as oat husks, not yet explored in limestone-based composites, as more sustainable alternatives for use as novel aggregates may be improved through the adoption of well-known valorisation strategies applied to other plant-based resources. In this context, this work innovates by assessing how treatments on oat husk surfaces and the choice of limestone-based binders impact the mechanical performance of composites. The strategy adopted to achieve these objectives, in addition to carrying out the physical and geometric characterisation of the husks, consists of treating the husks’ surface using washing cycles in water, cement/pozzolan binder, and linseed oil. Furthermore, matrices combining cement, calcium hydroxide Ca(OH)_2_, and microsilica (SiO_2_) were used. In conclusion, even though the effects of different binder combinations are inconsistent, coating oat husks—especially with linseed oil—works well in delaying particle degradation and improving mechanical strength compared to untreated particles. Furthermore, when aggregates are substituted with the longer and lamellar particles of oat husk, the impact of the water/cement ratio on mechanical performance and composite workability significantly decreases.

## 1. Introduction

The utilisation of limestone-based binders as construction materials has been prevalent throughout the evolution of human society, with Portland cement emerging as the most widely consumed globally, reaching 4.1 billion tons in 2020 [1]. Due to its hardening after hydration, it is often used to produce composites by forming a matrix in which dispersed particles adhere, with mortar and concrete being the best-known examples [2].

As a technological innovation in lime-based and cement-based composites, there is a growing interest in incorporating by-products made of cellulose, hemicellulose, and lignin—commonly referred to as lignocellulosic materials—especially from agricultural origin, as a more sustainable means of adding value to this material [3,4]. These efforts explore the circular use of resources and the utilisation of renewable and nature-based materials, such as plant-derived materials, to counterbalance carbon emissions and minimise the environmental impact associated with the production of conventional construction materials [5,6].

Researchers worldwide are examining the potential of the unique properties of lignocellulosic by-products to enhance composite characteristics, such as thermal and acoustic conductivity, by incorporating them as alternative fillers or aggregates [7,8]. Simultaneously, using natural fibres addresses the tendency of cement composites to crack and shrink while reinforcing them by providing tensile strength and ductility at a lower cost, reduced weight, and less detrimental environmental effects when compared to steel or synthetic alternatives [9].

Among agricultural by-products, oat husks hold significant potential when incorporated as aggregates in limestone-based matrices. This innovative approach not only addresses the challenge of managing agricultural waste but also aligns with current research efforts aimed at sustainable resource utilisation [10].

Oat husks, a by-product of oat grain dehulling (*Avena sativa* L.), remain an underutilised resource, with Europe contributing 60% of the global grain production of 25.18 million tons, yielding a significant volume during dehulling, often with an undefined destination and commonly disposed of or buried [11,12,13]. However, despite their underutilisation, these husks have promising potential, yet to be thoroughly studied within the scientific community, to be a sustainable resource in the construction industry.

Some studies have examined their potential utilisation as a biochar, acting as a modifier in asphalt binders [14], as well as their viability for cellulose extraction [15] and as a partial substitute in particleboard manufacturing [16]. However, studies investigating the use of oat husks in cementitious matrices are still scarce, aside from recent efforts to explore their available silica as a potential natural pozzolan [17,18] and investigations about the effects of the extractives present in the husks on cement hydration [10], opening possibilities for the use of husks as aggregates.

As with other plant-based derivatives, challenges to their adoption as a construction material include the heterogeneity in plant composition [19,20] and the degradation suffered in an alkaline environment [2,21,22]. Therefore, addressing these challenges necessitates exploring integration strategies previously investigated in other agricultural resources to make using lignocellulosic materials viable.

One such strategy is particle pre-treatment, which aims to enhance compatibility with the matrix and of which there are different practices [9]. One of the well-known practices is hornification, a treatment that seeks to modify the structure and morphology of particles through drying and saturation cycles by immersion in water [9], aimed at improving dimensional stability [23], increasing tensile and bond strength [24], and reducing hydrophilicity through the creation of internal hydrogen bonds [25]. When applied to oat husks, this method may also remove water-soluble extractives on the surface of the particles [10].

A second pre-treatment strategy studied is coating the particles to promote a physical barrier between the particle and the matrix, attenuating the affinity with water, innate from the presence of hemicellulose and cellulose in the composition of plants [10], limiting exposure to the alkaline environment, and consequently, the degradation and leaching of the components present in its composition [26,27]. As observed in silane coating treatment, modifying the contact interface between the matrix and the material layer deposited on the particles’ surface can also favour its mechanical interlocking structure [28]. There are also several coating methods; however, some authors have investigated using oils and mineral binders [27].

While coating with linseed oil [27], a fatty acid oil, and stearic acid [29], a component of its composition, have demonstrated favourable results, improving its compatibility with the cementitious matrix [27,29], some authors have also investigated the pre-exposure to an alkaline environment [9,29], even similar to that found in the cement matrix, by coating the particles using Portland cement and pozzolanic agents [27].

The third strategy studied to improve plant-based particles’ suitability for use in concrete or mortar is to reduce the effects of alkaline degradation by using binders and mixtures based on non-hydraulic limes, a material that hardens only by controlled drying and the reaction of the present Ca(OH)_2_ with carbon dioxide (CO_2_) [30]. Using this material, mixtures are made with silicon dioxide (SiO_2_), forming calcium silicate (C–S–H) [10], the same agent responsible for the mechanical capacity of Portland cement. Blends with Portland cement and natural hydraulic lime (NHL) were also studied [31,32], promoting the partial hydraulic capacity of the mixture, that is, the ability to set in the presence of water [30].

Beyond considerations regarding aggregate/matrix compatibility, several factors affect the behaviour of composites in the fresh and hardened state, among them characteristics inherent to the particles, such as texture and geometry [33]. Mineral aggregates’ irregular shape, characterised by a large specific surface area and high surface energy, contributes to suboptimal flow properties in fresh concrete [34], while elongated and flaky particle shapes diminish compressive strength [35].

In summary, this study contributes to the global efforts in utilising lignocellulosic resources as an alternative and sustainable source for construction materials. This study innovates by addressing the absence of research on the use of oat husks, an abundant resource in Europe, yet largely unexplored as aggregates in limestone-based matrices, particularly those incorporating Portland cement. It also innovates by applying strategies already utilised in the study of natural fibres and composites, such as pre-treatments and matrix modifications, to enhance the compatibility between materials. This pioneering endeavour not only expands the spectrum of sustainable building materials but also underscores the importance of utilising regional, readily available, and underutilised resources for environmental and economic sustainability.

In specific, the objective of this study is to discern the influence of oat husk surface treatments and various limestone-based binders on the mechanical performance of composites, thereby bridging the existing gap in research concerning their viability as aggregates within limestone-based matrices.

Expanding upon preceding inquiries into the effect of extractives on cement matrices, the study examines the physical characteristics of oat husks as particles. Additionally, it evaluates the effectiveness of the selected surface treatments and the concurrent utilisation of diverse binders.

## 2. Materials

### 2.1. Oat Husks

**Figure 1:**
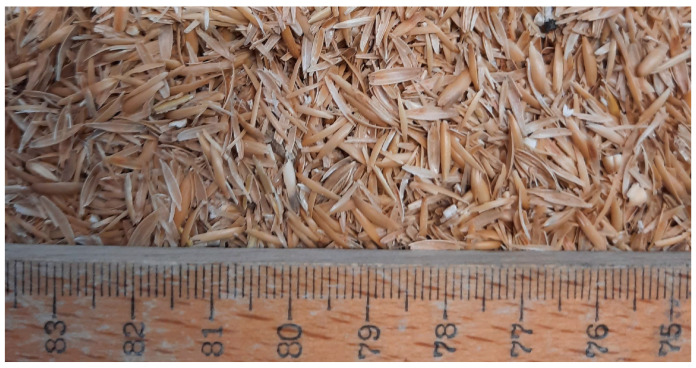
Oat husks after sieving.

As presented in Figure 1, the bio-aggregate utilised comprised oat husks collected in the Co. Wexford region of Ireland in July 2020, sourced from the materials previously examined by Bonifacio and Archbold [10,17].

To characterise the husks in their received condition, the sampling and quartering procedure recommended for hemp shivs by RILEM TC 236-BBM was applied [36]. The particles passing the 0.5 mm sieve were excluded from use. The granulometric distribution of the aggregates in their original condition was determined using the dry sieving method as outlined in EN 933-1 [37], employing sieves with square openings (d) and the diagonal of this opening (2^1/2^d) as recommended by RILEM TC 236-BBM [36].

### 2.2. Binders

This study employed four materials to fabricate the two distinct matrices under investigation.

To produce a cementitious matrix, Rapid Hardening Portland cement (RHPC), classified as CEM-I 42.5 R (supplied by Irish Cement Ltd., Platin, Drogheda, Ireland) by the EN 197-1:2011 Standard [38], was utilised due to its minimal secondary additives. For the production of other lime-based matrices, commercial calcium dihydroxide (Ca(OH)_2_), commonly known as hydrated lime (White Rhino Lime supplied by Clogrennane Lime Ltd., Clogrennane, Ireland), was employed in combination with natural hydraulic lime class 5 (NHL 5 provided by Secil Martingança S.A, Maceira, Portugal) in compliance with EN 459-1 [39].

Finally, silicon dioxide (>95% amorphous), commercially available as microsilica (Elkem Microsilica, Elkem ASA, Kristiansand, Norway), was utilised as a supplementary cementitious material (SCM).

### 2.3. Admixtures

In the treatment of bio-aggregates, raw linseed oil (Rustins Ltd., London, UK), with a supplier-stated relative density of 0.9 g/cm^3^ and a pH of approximately 7, was applied to augment the durability and resistance of the aggregates. To expedite the curing process, a liquid non-chloride accelerator (<0.1% chloride), specifically a calcium nitrate-based product (Arc Frostproofer & Rapid Hardener, supplied by Arc Building Products, Arklow, Ireland), was employed. The producer reports that this accelerator has a 1.2 g/cm^3^ density and a pH of approximately 10.

### 2.4. Mineral Aggregates

The mineral aggregate employed in this investigation was class 1 sand [40], exhibiting a fineness modulus of 2.70 and the fines content of 2.35%.

## 3. Oat Husk Particle Surface Treatments and Performance Evaluation Procedures

### 3.1. Surface Treatments

As part of the investigation, oat particles underwent surface treatment procedures. However, the untreated oat husks were designated as control oat husks (COHs).

#### 3.1.1. Cold-Water Washing Treatment

The cold-water washing treatment involved immersing oat husks in distilled water at room temperature for 24 h, draining the material thoroughly, and repeating the process for 2 cycles. This method aims to remove extractives such as saccharides and uronic acid, aligning with the findings from previous studies [10,27,41]. The specimens subjected to this treatment are denoted as washed oat husks (WOHs).

#### 3.1.2. Mineral Binder Coating Treatment

The mineral binder coating treatment entails coating oat husks with a grout formed by mixing microsilica and cement CEM I using a 1:1 mass ratio. Prepared with a binder/water mass ratio of 1 and a fibre/binder ratio of 2/3, this treatment aims to mineralise fibres partially and form a rough granular layer on the husks’ surface, serving as a barrier to the alkaline environment. The samples undergoing this treatment are labelled as pozzolan oat husks (POHs).

#### 3.1.3. Linseed Oil Coating Treatment

The linseed oil coating treatment involves covering the oat husks with a film formed by linseed oil, which undergoes hardening upon contact with air due to the autoxidation of unsaturated fatty acids in its composition [42]. This treatment, proven effective in limiting extractive leaching and isolating the fibre from the alkaline environment of the matrix [29,43], is executed by initially drying the oat husks at 50 ± 0.1 °C, followed by mixing them with linseed oil in a planetary mixer for 3 min using an oil/fibre ratio of 0.25 in mass, and subsequent drying at 50 ± 0.1 °C for 14 days [27]. The husks subjected to this treatment are named linseed oat husks (LOHs).

### 3.2. Treatment Confirmation

To visualise the characteristics of the untreated particles and confirm the applied treatments to the oat husks’ surface, a random particle from each sample underwent observation using a digital microscope in combination with a motorised Focusing Stand Controller (P-MFSC) and Touch Panel Monitor (P-TPM) (ShuttlePix P-400R, Nikon, Tokyo, Japan).

For further confirmation of the surface treatments, a random particle for each treatment was fixed using a compression force of 85 N in a universal attenuated total reflection (ATR) sampling accessory. Subsequently, four scans were conducted using an infrared spectrometer (PerkinElmer Spectrum One, Waltham, MA, USA) at room temperature, covering the spectral range from 650 to 4000 cm^−1^. The acquired data were analysed using a specialised software (Spectrum 10 software).

Scanning electron microscopy was employed to observe the particles’ surface details. The procedure for visualising the samples began by coating the particles with gold using a sputter coater (Bal-tec SCD 005, BAL-TEC AG, Balzers, Liechtenstein). The coated samples were affixed to a double-coated carbon conductive tab positioned on the top of the specimen stub pin and examined under a scanning electron microscope (TESCAN MIRA, Oxford Instruments, Cambridge, UK). The examination parameters included a view field of 2.17 mm, a magnification of 100×, a voltage of 10.00 kV, and a back-scattered electron detector (BSE).

### 3.3. Mass Variation after Immersion in Water

The variation in mass after water immersion was measured to assess the potential of the oat husk particles to leach extractives when immersed in an aqueous solution.

For each treatment, 2 g of the treated husks were dried in an electric oven at 60 °C for 42 h, immersed in distilled water for 24 h, and filtered under a vacuum using filter paper. The filtered samples were dried in an electric oven at 60 °C for 42 h before cooling in a desiccator and weighing on a tabletop scale (PCE-BSK 310, PCE Instruments, Manchester, UK). The material lixiviated was defined as the percentage difference between the material’s original mass (2 g) and the mass of the dried and cooled husks.

### 3.4. Modifications after Immersion in Alkaline Solution

To simulate the isolated effect of the alkaline environment characteristic of limestone-based matrices on the particles, a 1 mol/L NaOH solution was prepared using distilled water at 20 °C to achieve a pH of 14. The pH was measured using a pH test strip, which measures in intervals of 1, ranging from 0 for extremely acidic to 14 for highly alkaline, beyond the typical pH 12–13 found in cementitious mixtures [44], as a way to escalate the environmental stressors and assess the material’s resilience under harsher environments.

For each treatment, in quadruplicate, 1 g of the particles, dried in an electric oven at 60 °C over 42 h, was mixed in test tubes with 10 mL of the NaOH solution.

After 28 days, the pH of each sample was measured using a pH test strip, and the samples were neutralised (reaching pH 7) by rinsing them with distilled water and subsequent vacuum filtration using filter paper. The filtered samples were then dried in an electric oven at 60 °C for 42 h and cooled in a desiccator before being weighed using a benchtop scale (PCE-BSK 310, PCE Instruments, Manchester, UK).

The mass change was determined as the percentage difference between the initial mass of the dried husks (1 g) and the mass of the husks that had dried and cooled after exposure.

The previously dried particles were observed using the same digital microscope mentioned in Section 3.2 to visualise the changes in the samples exposed for 28 days.

### 3.5. Particle/Matrix Interference

#### 3.5.1. Assessment of Cement Setting Time Utilising Leachate Solutions

To investigate whether the material leached or transported from the particles affects the cement’s setting time, the Vicat setting time test was conducted using the Automatic Vicat test instrument (Controls Vicamatic 3 63-L2701, Hertfordshire, UK), following the guidelines outlined in EN 196-3 [45] but using a plaster paddle mixer with an electric hammer drill (Black+Decker 710 W, Leinster, Ireland) controlled by a generic external speed controller.

For sample preparation, the mixing water containing extractives was prepared by soaking the oat husks in a solution at 20 °C for 24 h using a liquid/mass ratio of 1:10, and filtering through a 0.25 mm mesh, similar to previous studies [10]. Distilled water was used for the control sample.

#### 3.5.2. Modifications after Use in the Cement Matrix

Additionally, random particles were extracted directly from the samples after conducting mechanical tests to assess the particle modifications and persistence of the surface treatment on the particles after use in the cement matrix. These particles underwent observation with digital microscopy and FT-IR analysis under the conditions presented in Section 3.2.

### 3.6. Aggregates Physical and Morphological Characterisation

#### 3.6.1. Bulk Density

The bulk density was determined per the guidelines proposed by RILEM TC 236-BBM [36], assessing the dry mass of the specimens contained within a cylindrical glass mould compared to the equivalent volume of distilled water.

The determination of the relative density of the oats samples (*γ*oat) was carried out in quintuplicate utilising a benchtop scale (PCE-BSK 310, PCE Instruments, Manchester, UK) in conjunction with a standard 20 mL (Vpyc) pycnometer calibrated considering water density (γw = 0.997 g/mL). The measurement was accomplished considering Equation (1), where the pycnometer’s initial mass (Mini), the mass of the pycnometer when filled with a known quantity of the sample (Msam), and the mass of the set when filled with water (Mwat).

*γ*oat = (Msam − Mini)/[Vpyc − (Mwat − Msam)/γw] (1)

#### 3.6.2. Water Content

The procedure for evaluating the water content of the aggregates was based on the guidelines of RILEM TC 236-BBM [36], using the evaluation quintuplicate of the initial mass and after drying in an electric oven at 60 °C over 24 h. The determination of water absorption after 24 h followed the established protocol, except for starting by immersing the aggregates in distilled water at room temperature for 24 h instead of drying the material and subsequent immersion, as specified. All the procedures were also applied to characterise the dry sand.

#### 3.6.3. Geometrical Analysis

To assess the cross-sectional dimensions of the bio-aggregates, 500 specimens from each treatment were measured using a standard digital calliper. The geometric attributes were subsequently determined by analysing over 500 particle samples that, to avoid bias, were randomly deposited on the surface of a conventional flatbed scanner and carefully arranged to prevent overlap, scanned at a resolution of 800 DPI using a conventional scanner against a black background (cover open), stored as Tagged Image File Format (TIFF), and analysed using a specialised software (ImageJ version 1.54f Java), similarly to procedures suggested by RILEM TC 236-BBM [36].

The area detection was set to only areas greater than 0.5 mm^2^ to avoid dust and segregated particles. The average particle volume was estimated and calculated as the product of the observed area and transversal section.

**Figure 2:**
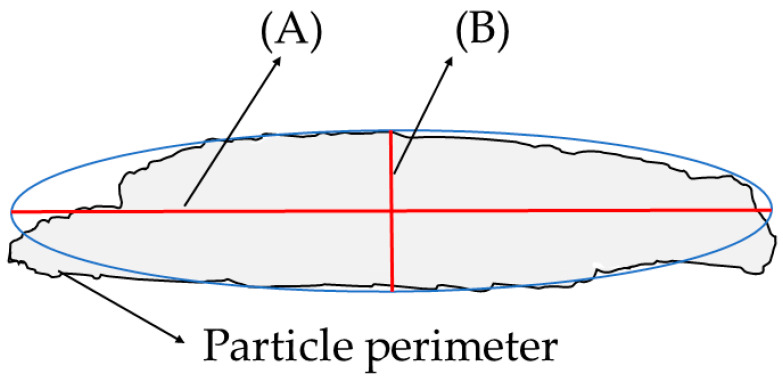
Elliptical representation of particle analysis: The horizontal red line represents the major axis (**A**), and the vertical red line represents the minor axis (**B**).

The particle analysis tool and built-in IJBlob [46] image library in ImageJ were used to determine the particle area and shape descriptors, considering circularity as 1 for a perfect circle, and the lower the values, the higher the existence of changes in area or perimeter, indicating an increasingly elongated particle shape [47]. Aspect ratio was defined as the ratio of the major and minor axis lengths of an approximate ellipse as Figure 2, with zero indicating a perfect circle and increasing as deformation increases. Lastly, roundness is a measure of the sharpness of the surface perimeter, with 1 reflecting the lack of surface imperfections [47] (corners and edges) and lower values indicating more sharpness of angular convexities and concavities.

### 3.7. Considerations for Statistical Acceptance of Particle Analysis Results

Aggregate characterisation adhered to the criteria outlined in RILEM TC 236-BBM [36], which defines results with a coefficient of variation (C.V) below five as valid; the same criteria were adopted for the dimensional analysis of the particles and calculation of shape factors. The confidence interval (C.I) was calculated using the two-tailed Student’s T-distribution with a 95% confidence level and degrees of freedom equal to the number of samples minus one.

## 4. Results and Discussion on Particle Surface Treatments and Performance Evaluation

### 4.1. Surface Treatment Confirmation

**Figure 3:**
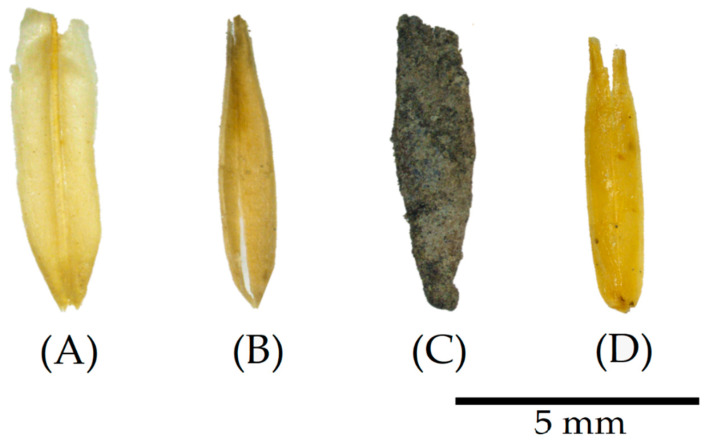
Digital microscopy of particles after treatment. (**A**) Untreated, control oat husks (COHs), (**B**) washed oat husks (WOHs), (**C**) pozzolan oat husks (POHs), and (**D**) linseed oat husks (LOHs).

The images presented in Figure 3, captured through digital microscopy, demonstrate the apparent effects of the different treatments on the dried oat husks. Initially, the untreated oat husks (COHs) exhibit a relatively flat surface with visible grooves and opaque colour. However, after washing cycles (WOH), the husks retain grooves but display a subtle darkening in colour. A minor axis shrinkage is also observable, characterised by a slight curl around the central axis, emphasising the husk’s natural concavity.

The alterations become more pronounced in the particles subjected to the surface material deposition treatments. For instance, the coating with a binder (POH) reveals a rocky appearance due to its greyish colour and rough surface. Similarly, linseed oil (LOH) deposition is apparent through its shiny aspect, forming a smooth and translucent layer over the particles’ characteristic grooves.

When the particles are submitted to FTIR analysis, the effectiveness of the treatments is evidenced through the changes in the spectra obtained compared to the sample without the treatments (POH).

**Figure 4:**
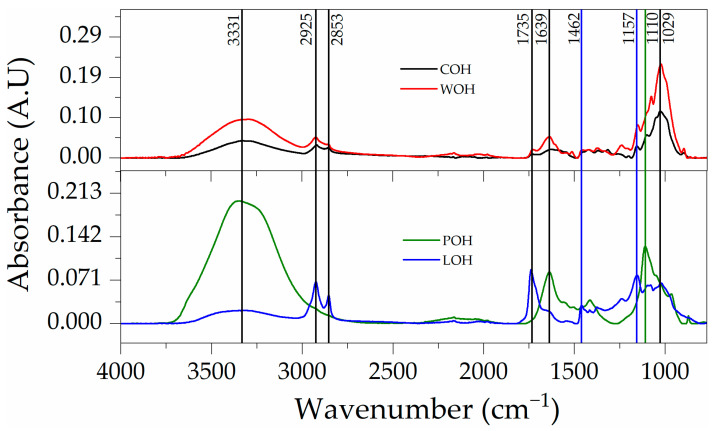
FTIR spectra of oat husks subjected to surface treatment.

Observing Figure 4, it is evident that in the samples subjected to washing (WOH), the pattern in the absorbed wavelengths was maintained, demonstrating an increase in absorbance. In the spectra of the samples subjected to coating (POH and LOH), despite some coincidences, they showed changes in intensity and absorbed wavelengths.

At 1735 cm^−1^, a minor signal peak in COH intensifies in WOH, indicating a potential aldehyde stretching ν(C=O) in hemicellulose and/or lignin [48], also with a sharp peak in LOH, related to linseed oil oxidation [49].

Centred at 1639 cm^−1^, the peak intensity rising in WOH, also present in POH when in conjunction with absorption at 3331 cm^−1^, characterises the bending vibration (v2) of the water molecule δ(O–H) [50].

In 1462 cm^−1^, a distinctive peak appears in LOH, signalling the presence of dried linseed oil δ(C-H) [49].

The increase in the intensity of peaks in the region of 1157 for WOH and LOH may be associated with the asymmetrical stretching ν_as(C–O) of cellulose and hemicellulose [51].

A noteworthy development occurs at 1110 cm^−1^, where a visible peak corresponds to SO_4_^2−^ vibration (v3) in sulphates observed in the hydrated cementitious samples ν(C–O) [52]. Finally, the peak at 1029 cm^−1^ may be associated with C–O, C–C, or C–OH bending in hemicellulose [53].

**Figure 5:**
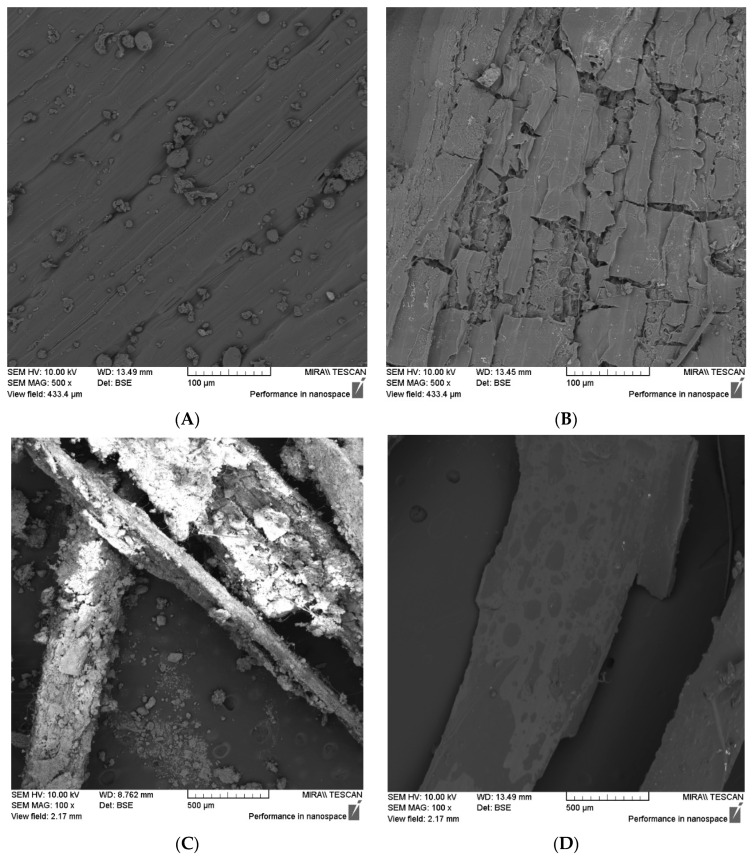
SEM images of oat husks after surface treatment under 500× magnification: (**A**) untreated, control oat husks (COHs) and (**B**) washed oat husks (WOHs), and under 100× magnification: (**C**) pozzolan oat husks (POHs) and (**D**) linseed oat husks (LOHs).

In terms of visually confirming the effectiveness and consequences of the surface treatments observed by the digital microscopy in Figure 3, the SEM confirms that the surface pattern in the untreated material (COH) in Figure 5A is characterised by oriented grooves arranged practically in the same plane. However, after undergoing washing cycles (WOH), as shown in Figure 5B, the surface pattern changed, indicating an increase in porosity, attributed to the fracture or rupture of the surface layers, possibly caused by the swelling effect following the successive washing and subsequent drying of the material. In Figure 5C, it is possible to see that the binder was deposited and agglomerated on the surface of the husks (POHs), forming irregular layers, different from the formation of a uniform film layer by coating the oat husks with linseed oil (LOHs), evident in Figure 5D.

In general, the experiments validate the efficacy of the execution of the surface treatment. The surface modification of the washed particles (WOHs) is likely linked to the anticipated removal of water-soluble substances, thereby promoting an initial increase in porosity and water accessibility within the particle.

Moreover, the heightened exposure of holocellulose (comprising both cellulose and hemicellulose) is discernible in the FTIR spectra, manifested by an augmented absorbance observed at wavelengths linked to these constituents. This increased exposure underscores the significance of the plant components and water interaction.

Another crucial aspect to consider is the facilitated interaction of the cellulose hydroxyl groups, renowned for their tendency to swell due to the arrangement of water molecules in semi-crystalline regions [54]. This interaction, combined with the sorption/desorption hysteresis behaviour [54] acting across various particle regions following forced drying, may have contributed to the observed cracking pattern and the curved aspect of the particles.

Regarding coating treatments, alongside the visual alterations noted, akin to those documented by previous studies [26,27,55], the divergence in the FT-IR spectrum from the pattern observed in the untreated material corroborates the efficacy of the treatment execution.

### 4.2. Particle Mass Analysis Post Immersion in Water

The decrease in the mass after immersion in water, as observed in Table 1, can potentially signify the removal of the water-soluble extractives within the particles.

**Figure 6:**
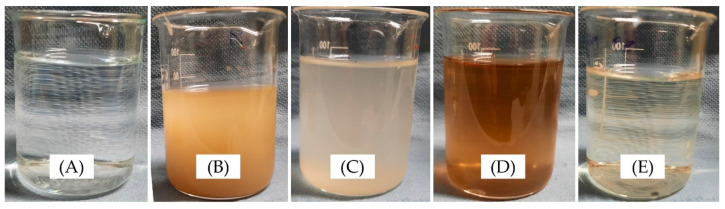
Filtered solution obtained after the immersion of the husks in water, indicating (**A**) pure water as control, (**B**) COH, (**C**) WOH, (**D**) POH, and (**E**) LOH.

The mass reduction observed in the WOH samples aligns with the anticipated quantities post-treatment, contributing to the 88% differential mass removal compared to COH. However, observing the leached solution in Figure 6, linking this phenomenon solely to removing extractives is imprecise in the coated particles. In the POH samples, the 39% variance might also be attributed to the removal of the deposited material; however, in LOH with the 78% difference, the hydrophobic nature of the coating implies the removal of extractives due to water percolation through treatment flaws.

### 4.3. Particle Modification after Immersion in Alkaline Solution

**Figure 7:**
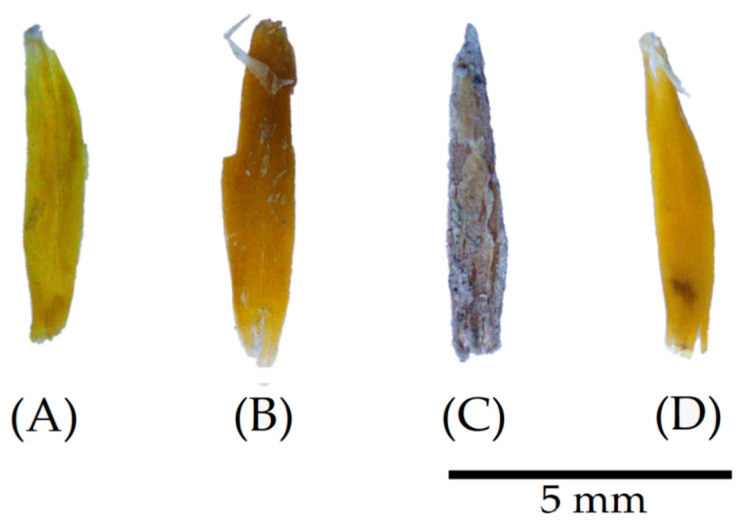
Digital microscopy of particles after 28 days of exposure to alkaline solutions. (**A**) Untreated, control oat husks (COHs), (**B**) washed oat husks (WOHs), (**C**) pozzolan oat husks (POHs), and (**D**) linseed oat husks (LOHs).

To observe the visible effects of an alkaline environment on the treated husks, Figure 7 was obtained through digital microscopy after the exposure of the particles for 28 days to an alkaline solution (pH 14).

In general, the particles maintained their structure and geometry. However, excluding POH, the particles presented a smooth and slightly translucent appearance, with barely visible grooves and darkened colour. Notably, in the WOH and LOH samples, remains of a film are visible, highlighting a possible layer of degraded plant tissue.

Considering the husks subjected to coating, the POH particles maintained their coverage despite the reduced thickness of the material deposited in some places, making it possible to visualise the particle under the binder layer. On the other hand, traces of linseed oil are not identifiable in the LOH particles, suggesting at least the partial removal of the film formed on the particle’s surface.

As anticipated, the results outlined in Table 2 reveal that exposing the treated husks to the alkaline solution led to a loss of particle mass. Generally, excluding POH, most mass loss occurred within the first seven days of exposure. As observed in previous studies [10], the pH was reduced during this period, reducing from 14 to 13.

For both exposure periods, the results indicate that the particles without coating (COH and WOH) experienced similar mass losses with a subsequent increase of 10% and 15%, respectively, after the seven days.

The evaluation of mass loss in the coated particles (POH and LOH) appears to be more closely associated with removing the material from the particle surface, subject to varying removal rates rather than the degradation of the treated particle’s plant tissues.

In the case of the POH samples, the mass loss in the initial seven days of exposure was lower than in the other samples but increased after 28 days, representing 94% of the total mass loss in the 21-day interval. Conversely, the LOH samples exhibited the most significant mass loss in the initial 7-day period, which continued to rise after 28 days of exposure, reaching a 30% increase over 21 days.

The reduction in mass observed after subjecting the husks (mainly COH and WHO) to a high-concentration NaOH solution is anticipated, as this method effectively removes hemicellulose and lignin while promoting cellulose crystallisation [9]. Typically, lower concentrations of NaOH (between 5% and 10%) are utilised for fibre pre-treatment to promote and measure cellulose stability [56].

When assessing the POH samples, the primary and delayed mass loss may be attributed to the gradual degradation of the coating under continuous exposure to the highly alkaline solution. However, only one study was identified regarding mass loss while investigating concrete immersed in a 10% NaOH solution, which reported a mere 0.7% and 1.87% mass loss after 7 and 28 days, respectively [57]. Additionally, combined with lignocellulose degradation, the mass loss may be attributed to superficial granule detachment caused by the loss of adhesion within the husk surface.

**Figure 8:**
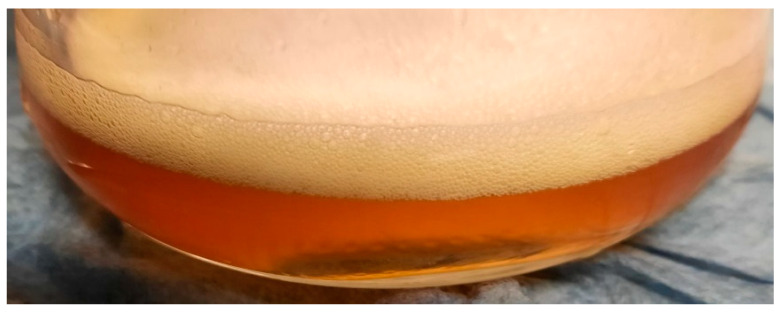
Filtered solution from linseed oil-treated particle exposed to NaOH solution.

Given that linseed oil can react with NaOH, the observed mass loss might also be linked to potential saponification, as evidenced by the formation of foam during particle filtration (see Figure 8), resulting from the possible hydrolysis of the triglycerides within the linseed oil composition [42,58], leading to the formation of fatty acid soaps and glycerol [58].

### 4.4. Particle/Matrix Interference Analysis

#### 4.4.1. Effect of Leachate Solution on Cement Setting Time

Regarding the effects of the leaching of the particle components in the matrix, Table 3 presents results indicating an increase in setting time across all the samples, with more significant variation observed in the time required to reach the final setting time. Despite the anticipated increase in setting time for the untreated samples [10] (COHs) reaching 32% and the washed samples (WOHs) reaching 21%, the coated particles exhibited even longer setting times, with POH and LOH showing setting times 48% and 38% longer than the control, respectively.

The literature establishes that the presence of silica may prolong the setting time of mixtures. However, considering that the surface treatment has already cured and hardened, one hypothesis for this delay is the potential leaching of extractives and degradation products suffered by the particles during coating. Nevertheless, upon observing the colour of the leached solution in Figure 6, the presence of other inhibitory elements may also contribute to the observed results.

The increase in setting time observed with the use of LOH highlights a possible presence of the substance in the solution, as evidenced by the solution’s colour in Figure 6 and by the known delaying effect of fatty oils on cement’s curing time [59].

#### 4.4.2. Particle Modification after Use in the Matrix

**Figure 9:**
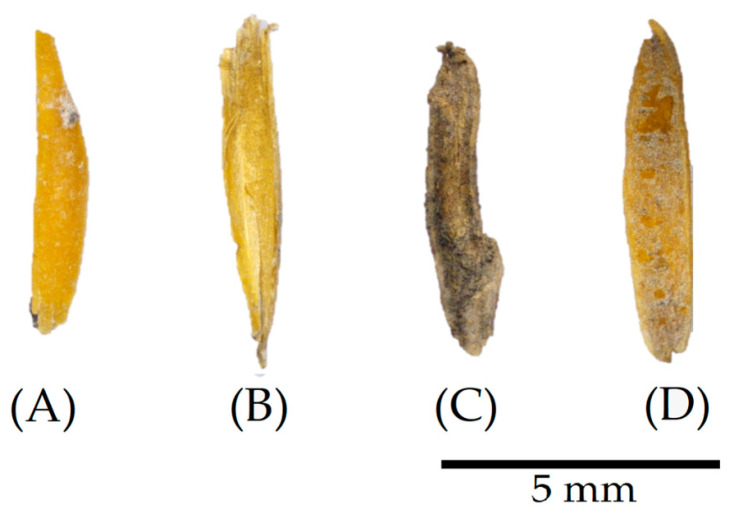
Digital microscopy of particles after 28 days of use in a cementitious matrix. (**A**) Untreated, control oat husks (COHs), (**B**) washed oat husks (WOHs), (**C**) pozzolan oat husks (POHs), and (**D**) linseed oat husks (LOHs).

In Figure 9, after utilisation in the cementitious matrix, it is possible to observe that the particles maintained their geometry yet exhibited similarities with those immersed in an alkaline environment (COH and WOH), revealing the removal of the layer formed after the coating treatment (POH and LOH).

The untreated particles (COHs) exhibited a smooth and slightly translucent appearance, with barely visible grooves and white marks indicating the removal of the outer epidermis + cuticle [60]. The washed particles (WOHs) exhibited minimal modification, but it is possible to identify less smooth contours, indicating degradation. Significant changes were observed in the particles subjected to coating, where (POH) appears to undergo a more substantial removal of the superficial layer, allowing the visualisation of the original particle. Similarly, the removal of the linseed oil film (LOH) and the deposition or possible encrustation of the matrix on the particle is evident.

**Figure 10:**
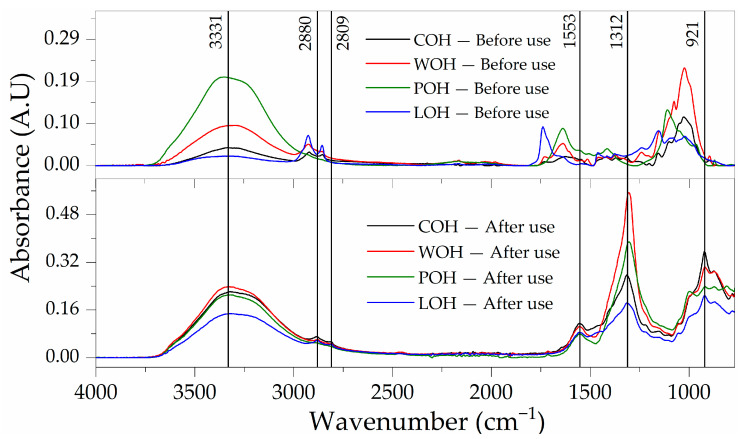
FTIR spectra of oat husks subjected to surface treatment before and after 28 days of use in cementitious matrix.

Examining in Figure 10 the samples’ FTIR spectra post-matrix interaction reveals a distinct pattern, with the peaks from each treatment disappearing. The samples now exhibit a consistent pattern, though with discernible differences in intensities.

The persistence of the peak in the 3331 cm^−1^ region, previously linked to ν(O–H) and the potential presence of water, remains noteworthy. The peaks in the region associated with alkanes ν(C–H) stretching exhibit reduced intensity and a shift to 2880 cm^−1^ and 2809 cm^−1^. A discernible peak emerges at 1553 cm^−1^, a region often correlated with amide bonds (C=O). However, the ν(NH) stretches associated with this functional group are absent in the 3200 cm^−1^ region.

In the “fingerprint” region, a strong peak at 1312 cm^−1^ is evident, possibly linked to the bending vibration of aliphatic groups δ(CH_3_ or CH_2_) or the presence of C–O groups. Finally, at 921 cm^−1^, a peak that may be attributed to various vibration modes, such as C–H in aliphatic groups or C–C in rings, is observed.

After use, the change in the spectral pattern of the particles in the FTIR analysis indicates that despite the treatments, the particles have undergone similar modifications on their surface. When associated with visual analysis, these modifications reveal particle degradation, presenting patterns similar to those observed after the exposure to the alkaline environment. Additionally, there may be surface encrustation and the removal of coating layers due to enhanced one-side adhesion with the matrix or, possibly, due to friction and the formation of ettringite bridges [2], which could increase mechanical adhesion.

### 4.5. Aggregate Granulometric Distribution and Bio-Aggregate Physical Characteristics

**Figure 11:**
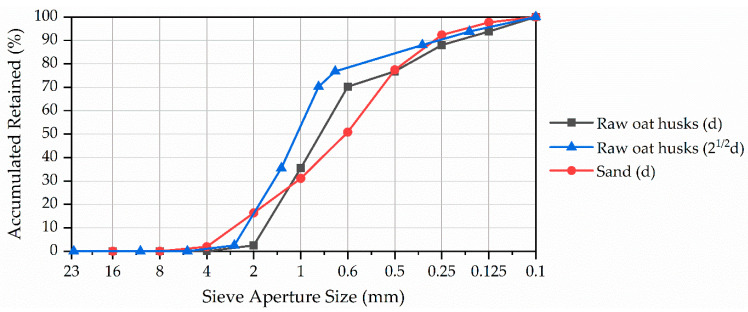
Particle size distribution of the aggregates using (d) as the edge of the sieve’s square aperture and retained percentual mass.

The raw oat husk sample exhibits, in Figure 11, a deviation from the uniform gradation pattern evident in the sand, concentrating the particles primarily between sieves with openings of 1 mm (33% retained) and 0.6 mm (34.75% retained). The granulometric analysis reveals that the fraction of oat husks employed (retained in 0.5 mm sieve) constitutes ~76% of the received material’s mass. When considering the diagonal measurement (2^1/2^d) of the square sieve opening, it is inferred that mostly the particles with a minimum dimension of ~0.71 mm were utilised.

When evaluating the dimensions of the particles, the average values expressed in Table 4 show that the cross-section of the particles represents approximately 2% of the major axis and 11.2% of the minor axis.

**Figure 12:**
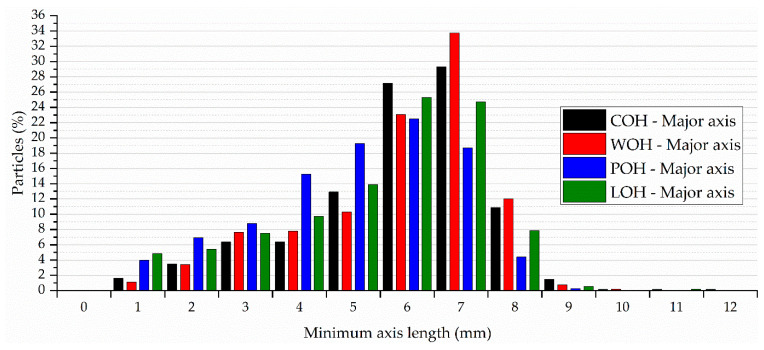
Particle distribution based on major axis length of equivalent ellipse.

**Figure 13:**
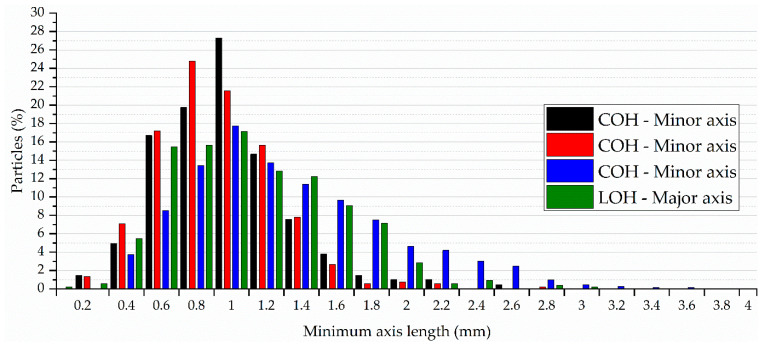
Particle distribution based on minor axis length of equivalent ellipse.

Associated with the distribution presented in Figure 12, there is a greater concentration of particles at values lower than the average value of the major axis. However, analysing the minor axis in Figure 13, it is observed that this distribution occurs after the average value. Both the figures indicate a greater distribution in the dimension of the axes in the samples treated with binder (POH) and subjected to linseed oil coating (LOH).

The evaluation of the particle shape descriptors, expressed in Table 5, indicates that despite the subtle change after the treatments, the particles generally present more angular or irregular contours with a significantly longer shape in one direction than the other, as confirmed by analysing the axes.

When consolidating the findings, despite the reduced cross-section of the oat husks, the granulometric analysis seems to provide a good estimate of the investigated particle sizes. When coupled with visual inspection, the particles’ geometric characteristics reveal a flat, elongated morphology even after treatment.

This shape increases the aggregate-specific surface area and, consequently, surface energy while reducing binder workability due to increased friction between the particles [34], decreased packing efficiency resulting from constraints in their arrangement within the matrix, and a consequent reduced compressive strength, as observed with natural aggregates [35]. Nonetheless, these characteristics may foster the formation of orderly stacking structures and subsequent layers within the matrix.

In terms of aggregate and bio-aggregate physical characteristics, the results in Table 6 show that, when considering the geometric properties of oat husks, their inherent tendency to retain their natural curved shape and known lower specific density [60] play a significant role in packing the particles and affecting the resulting bulk density.

When comparing the untreated material with the husks after the washing cycles (WOHs), the reduction in density is consistent with the increase in particle porosity. The same phenomenon can also be present in the material treated with a binder (POH), which has different amounts of material fixed to the surface of the husks, favouring the accommodation of the particles and increasing bulk density. However, when evaluating the material subjected to coating using linseed oil (LOH), the reduction in density may be associated with the air trapped below the oil layer and lower oil density [61], considering that the bulk density was also reduced. Regarding particle-specific density, the results demonstrate that oat husks have half the density of sand. However, the same volume of bulk material only represents, on average, ~85% of the mass of the mineral aggregate.

Also, in Table 6, the high percentage of initial moisture in the samples containing mineral particles may be associated with factors such as porosity, capillarity, and agglomeration, considering the particles’ visible size and surface area. The tubular internal structure of oat husks [2,60], associated with the increase in porosity, may also be related to the results observed in the washed husks (WOHs), in addition to greater exposure to chemical components with greater affinity with water, such as hemicellulose, since after immersion for 24 h, the sample absorbed almost twice as much water compared to the untreated material (COH). However, the lower values in the coated samples (LOHs), despite showing some absorption and being in line with the hydrophobic nature of the oil used, may suggest a failure in coating some particles.

## 5. Procedures for Mechanical Analysis of oat Husks as Aggregates in Limestone-Based Composites

### 5.1. Mixing Procedures and Sample Casting

To replicate the process usually used to prepare a cement paste with standard consistency [45], all mixtures were prepared using a plaster paddle mixer with an electric hammer drill (Black+Decker 710 W, Leinster, Ireland) controlled by a generic external speed controller.

The mixing process involved combining a sequence of aggregates, water, admixtures, and binders in a 5-litre bowl. Subsequently, the mixture was mixed using a rotation speed of 200∼250 rpm for 30 s, followed by a thirty-second pause to scrape the paste from the inner walls of the bowl and subsequent mixing at a rotation speed of 250~300 rpm for 60 s.

The mortar samples were made using the process cited and mixing proportions described in Section 5.2, Section 5.3 and Section 5.4. They were then moulded in 2 cm thick layers, tamped 32 times, crossed using a 10 mm diameter rod, and vibrated for 5 s in three 40 mm × 40 mm × 160 mm moulds, as specified in the EN 196-1 Standard [62]. The samples were kept in sealed polypropylene bags until demoulding at the test age (28 days).

### 5.2. Sample Design to Evaluate Surface Treatment Influence

To define the design, all proportions consider material bulk densities, and the additional water considers the water absorption observed after 24 h for each bio-aggregate, excluding WOH, which utilises the same value assigned to COH.

Equivalent material volumes were used in the samples without bio-aggregates (control).

### 5.3. Sample Design to Evaluate Types and Combinations of Binder Influence

To assess the impact of using the treated particles on the mechanical properties of composites, the samples were prepared in the proportions shown in Table 7, where the samples with aggregate replacement received the prefixes P_(partial replacement) and F_(full replacement). The choice of 33% replacement was adopted to emphasise the effect of the husks and minimise potential delays in curing, based on preliminary tests utilising 50% of COH, designated with the T_(test) prefix.

To assess the influence of aggregate replacement in different binders, the samples were prepared following the proportions shown in Table 8, receiving or not in the name, the prefix C_(control), P_(partial replacement) or F_(full replacement) and the primary binders and admixtures separated by an underscore (_). The proportions were based on the builders’ mix used by the authors studying hemp/lime concretes [63].

To evaluate the pH within the matrices, the samples that did not incorporate husks were turned into powder after 28-day curing and mechanical tests by crushing with pliers, mortar, and pestle and subsequent sieving through a 90 μm sieve.

Following that, a solution for each binder was formulated by combining 5 g of a sample and 10 mL of freshly distilled water at 22 ± 1 °C. After a one-minute stirring duration using a glass stirrer, the mixtures underwent vacuum filtration utilising filter paper grade 40 (Whatman, Fisher Scientific Ireland Ltd., Dublin, Ireland).

Post-filtration, the pH of the resulting liquid was measured using a pH probe and meter (SLS Lab Pro Hydron Benchtop pH Meter, Scientific Laboratory Supplies Ltd., Dublin, Ireland), adhering to the manufacturer’s instructions and calibration using suitable buffer solutions.

### 5.4. Sample Design for Assessing Mechanical Performance

To assess the mechanical behaviour of the samples with varying material proportions, the samples were designated with a naming convention.

Each sample is named by its water/cement ratio, abbreviated as W/C (A, expressed in percentage), aggregate replacement ratio (B, expressed in percentage), and additive/cement ratio (C, expressed in percentage), separated by underscores (_). The proportions for each sample are expressed in Table 9, with an aggregate/cement ratio of 2.08 in volume.

### 5.5. Fresh and Hardened State Mechanical Analysis Methods

The material prepared using the previously mentioned mixing methods and proportions was evaluated by applying the principle of spreading a sample after cycles of free fall of a platform, characterising an adaptation of the flow table consistency measurement method presented in EN 1015-3 [64] to assess the consistency and workability of the mortars in their fresh state.

The modified process involved employing a generic hand-operated flow table, adapting the use of a truncated conical mould utilised in the Vicat test described by EN 196-3 Standard [45], centring the cone on the previously moistened elevating platform, and moulding the samples following the suggested procedure detailed in EN 196-3 Standard [45]. After filling, levelling the top, and removing the mould using a steady upward pull movement, the platform was cyclically raised 12.5 mm and dropped 30 times in about 30 s, followed by diameter analysis by the average of six symmetrical measurements carried out using a digital standard calliper.

To make sample assessment possible, the spreading of each sample, named as slump, was compared in percentage terms to the scattering values observed in the samples subjected to the same procedure. The samples comprised a cement paste of standard consistency (named standard) measured following EN 196-3 Standard [45].

Following 28 days of curing, the samples were subjected to specific gravity assessment using an industrial specific gravity balance (Stable Micro Systems SG/15–395, England, UK). Subsequently, flexural and compressive strength analyses were conducted in accordance with EN 196-1 [62] guidelines. For the flexural test, a universal testing machine with a capacity of 300 kN (Instron 300DX-B1-G4-G1A, England, UK) was utilised, while a 2000 kN capacity compression machine (ELE ADR Touch SOLO 2000, ELE International, England, UK) was employed for the compression test. The results were reported as flexural strength (Rf, 28 days) and compressive strength (Rc, 28 days). Following that, the results were compared, and the percentage difference (C.D) was calculated concerning a control sample, which consisted of the standard consistency mortar (standard), samples named control, samples with the prefix C_, and (45 or 50 or 60)_0_0, respectively.

### 5.6. Considerations for Statistical Acceptance of Composite Strength

Acceptance criteria outlined by the American Concrete Institute [65] were applied for mechanical strength testing, which classifies laboratory samples as valid if their variability presents standard deviation (S.D.) values below 5.

## 6. Results and Discussion on Composite Mechanical Analysis

### 6.1. Surface Treatment Effect

The mechanical test results conducted using the treated husks indicate in Table 10 a reduction in workability when using the particles treated by washing cycles (WOHs) compared to the untreated particles (COHs), both in partial replacement (42%) and total replacement (20%). However, the coating treatments (POH and LOH) improved matrix behaviour by 22% and 31% in partial replacement and 65% and 48% in total replacement, respectively.

Similarly, the density observed in the samples subjected to partial replacement decreased for all the other samples (WOH, POH and LOH) compared to the samples containing the untreated particles (COHs). However, after the total replacement of the aggregates, both the POH and LOH samples showed an increase of 6% and 9%, respectively.

In mechanical results, adding the particles treated by coating (POH and LOH) improved compressive strength by 12% and 32% in partial replacement and 468% and 312% in the total replacement of the aggregates compared to the untreated particles (COHs). Furthermore, flexural tests surpassed the untreated particles, reaching 55% and 269% for POH and LOH after total replacement.

The reduction in workability may also be attributed to a possible decrease in free water due to the increased immediate absorption inherent to oat husks combined with the geometry of the particles [34]. However, despite the anticipated and observed decrease in density with the incorporation of bio-aggregates, the reason for the density variation among the different samples is not evident. This variation may be attributed to the amount of free water available or the air entrapment in the particles without coating (COH and WOH), which is reduced in the particles with a surface barrier (POH and LOH).

Regarding mechanical strength, the expected decrease with the incorporation of bio-aggregates may be linked to the lamellar nature of the particles, which could reduce particle packing and interlocking during compression [35]. Similarly, as observed with hemp, the compressive strength declined in LOH but differed by presenting better results than the untreated material [35]. However, the development of a low-quality interface, as observed, may be one of the factors contributing to the observed results [61].

Significantly, the difference in flexural strength observed in the composites using the particles subjected to coating (POH and LOH), coupled with visual evidence after use in the matrix, may be attributed to better anchoring, owing to increased friction between the particle and the matrix, as evidenced by the removal of the coating after mechanical tests. This enhanced adhesion contrasts with the behaviour anticipated in the uncoated material, favouring ettringite bridge formation [2] due to capillary water absorption.

The lower mechanical results observed in the WOH samples may also be related to a possible more intense particle degradation.

### 6.2. Binder Type Influence

The assessment of the matrices’ pH after twenty-eight days of hydration, as presented in Table 11, demonstrates that the samples exhibited a strong alkaline nature, showing no significant difference regardless of the evaluated binder combinations.

The evaluation of the different control samples of each binder, presented in Table 12, revealed that the binders containing Ca(OH)_2_ exhibited increased workability. However, this was accompanied by a reduction of more than half in density and around 95% in compression and flexion strength compared to the C_RHPC sample.

During the partial replacement of the mineral aggregates, when compared with their respective control samples, the samples demonstrated reduced workability and increased density, except for the P_RHPC_SiO_2_ sample, which exhibited a decrease in density.

The samples generally experienced a reduction in compression and flexural strength, except for the P_RHPC_Ca(OH)_2_ sample, which showed an increase in compressive strength, and the P_Ca(OH)_2__SiO_2_ sample, which exhibited a remarkable +10,372.6% increase in flexural strength, surpassing the values of the other samples.

Upon the total replacement of the aggregates, an apparent reduction in workability was observed, and the samples containing Ca(OH)_2_ were the only ones to present a higher density than the control samples. Compressive and flexural strength were uniformly reduced for all the samples except those without Ca(OH)_2_. Notably, the F_Ca(OH)_2__SiO_2_ sample exhibited increased flexural strength.

Regarding workability, the results align with the anticipated increase in the samples containing Ca(OH)_2_. In contrast, the reduction observed in the samples containing SiO_2_ may be attributed to the interaction between the particles and increased water demand, depending on the surface area of the SiO_2_ grains [66]. This behaviour could be exacerbated by the potentially heightened immediate absorption inherent to oat husks coupled with the particles’ geometry [34].

The factors contributing to the observed variations in density are not entirely clear. These could be associated with various factors, including the characteristics of the aggregates, the presence of air, the measurement method adopted, and the influence of densification of the composite’s crystalline structure due to the addition of SiO_2_ and Portland cement [66]. Similarly, the factors influencing mechanical strength are not entirely evident. They may be associated with the influence of binders and leached extractives [10] and the geometry affecting particle packing and interlocking when the composite is subjected to compression [35].

### 6.3. Results of Mechanical Tests on Composites in Fresh and Hardened States

The data presented in Table 13 show that the slump increases proportionally with the addition of water and setting accelerator additives. However, this characteristic exhibits a non-linear reduction based on the rise in the percentage of aggregate replacement, particularly asserting dominance with total replacement.

On the contrary, specific gravity tends to decrease inversely to the influencing factors, significantly impacted by the aggregates’ replacement. The increase in slump is expected due to the increase in the liquid component of the mixture, which tends to “separate” the particles, and the accelerator may act as a plasticiser, increasing the workability [67]. On the other hand, workability is also influenced by the particles’ lamellar geometry, which limits the mobility and arrangement of the grains [34].

In terms of compressive strength, there is a noticeable correlation where an increase in aggregate replacement results in a substantial and non-linear reduction, accompanied by a subtle decrease with the addition of water.

As expected, incorporating additives increases the observed values [67], with the addition of 12% demonstrating slightly higher values than those obtained with a 20% addition. Similarly, and more pronouncedly, flexural strength diminishes with aggregate replacement, yet it exhibits a subtle positive increase with the use of 12% additives and 50% water.

## 7. Conclusions

This study significantly contributes to our understanding of how various limestone-based binders and oat husk surface treatments influence the mechanical properties of composites after 28 days of curing. Our findings suggest that while the combination of binders does not consistently enhance mechanical performance or reduce matrix alkalinity, surface treatments—especially coating with linseed oil—can effectively delay particle degradation and improve mechanical strength compared to untreated particles within the 28 days investigated. However, this enhancement comes at the cost of longer matrix setting times.

Furthermore, as the replacement of aggregates with elongated and lamellar particles from oat husks increases, the influence of the water/cement ratio on the slump and the mechanical performance of the composites diminish significantly. Instead, the composite’s performance is influenced substantially by setting-accelerating additives and density, although traditional parameters remain predictive of its behaviour.

Some further implications may also be drawn from the study, including:

- Treatments can be effectively carried out on the particles;
- Surface coating (POH and LOH) reduces mass loss during water immersion but increases cement-setting inhibitors;
- Coating (POH and LOH) delays degradation in alkaline solutions but may lead to partial particle degradation and coating removal when used in cement;
- The particles maintain a flat, elongated geometry post-treatment, although their specific density decreases;
- Coating treatment decreases particle water absorption and enhances mortar workability;
- Incorporating a setting accelerator additive enhances composite mechanical behaviour.

## Figures and Tables

**Table 1:** Particle mass removal after immersion in water.

Sample	COH	WOH	POH	LOH
Mass removal (%)	15.44	1.85	9.38	3.40
S.D	0.0859	0.0618	0.3099	0.2590

**Table 2:** Particle mass removal after 28-day exposure to alkaline solution.

Sample	Exposure (Days)	Mass Removed (%)	Standard Deviation	Difference from COH (%)	pH
COH	7	31.2	1.090	0.00	13
WOH	7	31.6	1.116	1.21	13
POH	7	26.4	0.357	−15.54	13
LOH	7	38.1	0.846	22.17	13
COH	28	35.8	0.625	0.00	13
WOH	28	34.7	0.497	−2.89	13
POH	28	51.1	2.116	42.71	13
LOH	28	49.7	1.037	39.02	13

**Table 3:** Vicat test results for using the filtered solutions obtained after the immersion of the husks in water.

Leached Sample	Initial Setting Time	C.I	Final Setting Time	C.I
	(Hour:Minute:Second)	95%	(Hour:Minute:Second)	95%
Control	02:19:02	±00:14:21	02:36:59	±00:18:18
COH	02:39:21	±00:19:03	03:26:48	±00:10:32
WOH	02:23:11	±00:12:13	03:09:51	±00:06:48
POH	02:58:30	±00:19:06	03:51:50	±00:12:25
LOH	02:39:17	±00:10:12	03:35:57	±00:16:08

**Table 4:** Aggregate morphological characteristics.

Aggregate	Axis		Dimension		
	Major (mm)	Minor (mm)	Transversal (mm)	Area (mm^2^)	Theoretical Volume (mm^3^)
COH	6.413	1.077	0.128	5.487	0.138
WOH	6.408	1.033	0.115	5.267	0.119
POH	5.544	1.415	0.127	6.386	0.180
LOH	5.902	1.213	0.155	5.831	0.188

**Table 5:** Particle shape descriptors.

Aggregate	Shape Descriptors		
	Circularity (0 to 1)	Aspect Ratio (0 to ∞)	Roundness (0 to 1)
COH	0.261	6.514	0.185
WOH	0.261	6.796	0.178
POH	0.297	4.43	0.282
LOH	0.313	5.632	0.236

**Table 6:** Physical characteristics of aggregates.

Aggregate	Bulk Density		Specific Density		Initial Moisture		Water Absorption after 24 h	
	kg/m^3^	C.V	ratio	C.V	%	C.V	%	C.V
Sand	1853.17	3.94	2.56	1.11	15.72	4.53	12.30	0.73
COH	236.94	3.53	1.268	3.29	8.36	0.84	57.22	2.68
WOH	235.67	2.09	1.238	2.75	14.71	0.21	103.84	2.70
POH	373.13	0.97	1.219	2.95	44.32	0.36	37.50	3.76
LOH	230.04	1.26	1.123	2.84	8.41	0.22	10.511	1.402

**Table 7:** Composition of samples with treated particles.

Sample	Husk	Sand	COH	WOH	POH	LOH	CEM I	Water	Additional Water	Aggregates /Binder
	%	kg/m^3^	kg/m^3^	kg/m^3^	kg/m^3^	kg/m^3^	kg/m^3^	kg/m^3^	kg/m^3^	ratio
Control	0	1252	0	0	0	0	417	209	0	3.0
T_COH	50	626	80	0	0	0	417	209	46	1.7
P_COH	33	839	53	0	0	0	417	209	30	2.1
P_WOH	33	839	0	53	0	0	417	209	30	2.1
P_POH	33	839	0	0	83	0	417	209	31	2.2
P_LOH	33	839	0	0	0	51	417	209	5	2.1
F_COH	100	0	160	0	0	0	417	209	92	0.4
F_WOH	100	0	0	159	0	0	417	209	91	0.4
F_POH	100	0	0	0	252	0	417	209	95	0.6
F_LOH	100	0	0	0	0	155	417	209	16	0.4

**Table 8:** Composition of samples with different binders and untreated particles.

Sample	Husk	Sand	COH	CEM I	SiO_2_	Ca(OH)_2_	NHL 5	Water	Additional Water	Aggregates /Binder
	%	kg/m^3^	kg/m^3^	kg/m^3^	kg/m^3^	kg/m^3^	kg/m^3^	kg/m^3^	kg/m^3^	ratio
C_RHPC	0	1252	0	417	0	0	0	209	0	3.0
C_RHPC_SiO_2_	0	1252	0	376	24	0	0	209	0	3.1
C_RHPC_Ca(OH)_2_	0	0	0	129	0	370	167	644	0	0.0
C_Ca(OH)_2__SiO_2_	0	0	0	0	73	370	167	644	0	0.0
P_RHPC	33	1252	0	417	0	0	0	209	30	2.1
P_RHPC_SiO_2_	33	839	53	376	24	0	0	209	30	2.2
P_RHPC_Ca(OH)_2_	33	0	53	129	0	370	167	644	30	0.1
P_Ca(OH)_2__SiO_2_	33	0	53	0	73	370	167	644	30	0.1
F_RHPC	100	0	160	417	0	0	0	209	92	0.4
F_RHPC_SiO_2_	100	0	160	376	24	0	0	209	92	0.4
F_RHPC_Ca(OH)_2_	100	0	160	129	0	370	167	644	92	0.2
F_Ca(OH)_2__SiO_2_	100	0	160	0	73	370	167	644	92	0.2

**Table 9:** Composition of samples for evaluating correlations between variables.

Sample	Sand	COH	CEM I	Water	Additional Water	Additive
A_B_C	kg/m^3^	kg/m^3^	kg/m^3^	kg/m^3^	kg/m^3^	kg/m^3^
45_0_0	1252	0	417	188	0	0
45_0_12	1252	0	417	138	0	50
45_0_20	1252	0	417	104	0	83
45_50_0	626	80	417	188	46	0
45_50_12	626	80	417	138	46	50
45_50_20	626	80	417	104	72	83
45_100_0	0	160	417	188	92	0
45_100_12	0	160	417	138	91	50
45_100_20	0	160	417	104	144	83
50_0_0	1252	0	417	209	0	0
50_0_12	1252	0	417	159	0	50
50_0_20	1252	0	417	125	0	83
50_50_0	626	80	417	209	46	0
50_50_12	626	80	417	159	46	50
50_50_20	626	80	417	125	72	83
50_100_0	0	160	417	209	92	0
50_100_12	0	160	417	159	91	50
50_100_20	0	160	417	125	144	83
60_0_0	1252	0	417	250	0	0
60_0_12	1252	0	417	200	0	50
60_0_20	1252	0	417	167	0	83
60_50_0	626	80	417	250	46	0
60_50_12	626	80	417	200	46	50
60_50_20	626	80	417	167	72	83
60_100_0	0	160	417	250	92	0
60_100_12	0	160	417	200	91	50
60_100_20	0	160	417	167	144	83

**Table 10:** Results of mechanical tests incorporating treated particles.

Sample	husk	Slump			Specific Gravity			Rc, 28 Days			Rf, 28 Days		
	%	mm	C.D (%)	S.D	g/cm^3^	C.D (%)	S.D	MPa	C.D (%)	S.D	MPa	C.D (%)	S.D
Standard		137		0.202									
Control	0	141	+3.3	2.621	2.19		0.007	48.59		2.720	10.28		0.685
T_COH	50	144	+5.3	1.966	1.71	−21.9	0.003	9.96	−79.5	0.705	3.67	−64.3	0.464
P_COH	33	132	−3.4	2.621	3.87	+76.4	1.715	11.19	−77.0	0.687	4.15	−59.6	0.389
P_WOH	33	77	−43.9	1.099	2.75	+25.6	0.037	10.60	−78.2	0.644	3.60	−65.0	0.384
P_POH	33	161	+17.6	3.807	3.41	+55.5	1.289	12.55	−74.2	0.829	4.37	−57.5	0.189
P_LOH	33	173	+26.5	3.490	3.26	+48.6	0.702	14.79	−69.6	0.911	4.08	−60.3	0.291
F_COH	100	106	−22.1	1.291	1.30	−40.9	0.023	0.35	−99.3	0.051	0.26	−97.5	0.068
F_WOH	100	85	−37.7	0.617	−0.33	−115.3	0.015	0.26	−99.5	0.047	0.09	−99.1	0.050
F_POH	100	176	+28.7	4.052	1.38	−37.1	0.376	2.01	−95.9	0.242	0.40	−96.1	0.376
F_LOH	100	158	+15.5	3.519	1.41	−35.7	0.038	1.46	−97.0	0.233	0.96	−90.7	0.038

**Table 11:** Matrix pH after 28-day curing period.

Sample	RHPC	RHPC_ SiO_2_	RHPC_Ca(OH)_2_	Ca(OH)_2__SiO_2_
pH	12.29	12.45	12.48	12.47

**Table 12:** Results of mechanical tests of untreated husks in varied matrices.

Sample	Husk	Slump			Specific Gravity			Rc, 28 Days			Rf, 28 Days		
	%	mm	C.D (%)	S.D	g/cm^3^	C.D (%)	S.D	MPa	C.D (%)	S.D	MPa	C.D (%)	S.D
Standard	0	137		0.202									
C_RHPC	0	141	+3.3	2.621	2.19		0.007	48.59		2.720	10.28		0.685
C_RHPC_SiO_2_	0	113	−17.4	1.341	2.12	−3.2	0.007	53.64	+10.4	1.276	9.16	−10.9	0.744
C_RHPC_Ca(OH)_2_	0	212	+55.0	2.796	1.05	−52.3	0.026	0.82	−98.3	0.047	0.60	−94.1	0.052
C_Ca(OH)_2__SiO_2_	0	185	+35.2	1.056	1.09	−50.3	0.014	1.10	−97.7	0.065	0.05	−99.5	0.024
P_RHPC	33	132	−3.4	2.621	3.87	+76.4	1.715	11.19	−77.0	0.687	4.15	−59.6	0.389
P_RHPC_SiO_2_	33	98	−28.1	0.466	2.04	−4.1	0.270	20.35	−62.1	1.574	4.84	−47.2	0.573
P_RHPC_Ca(OH)_2_	33	105	−23.2	0.850	1.42	+35.2	0.015	0.91	+10.1	0.052	0.16	−74.2	0.008
P_Ca(OH)_2__SiO_2_	33	100	−26.9	0.490	1.56	+43.2	0.037	0.45	−59.4	0.047	5.06	+10,372.6	0.552
F_RHPC	100	106	−22.1	1.291	1.30	−40.9	0.023	0.35	−99.3	0.051	0.26	−97.5	0.068
F_RHPC_SiO_2_	100	82	−40.2	0.493	1.44	−32.0	0.054	0.36	−99.3	0.047	0.27	−97.1	0.084
F_RHPC_Ca(OH)_2_	100	91	−33.3	0.446	1.11	+6.1	0.029	0.23	−72.2	0.051	0.13	−78.8	0.006
F_Ca(OH)_2__SiO_2_	100	85	−37.9	0.535	1.38	+26.2	0.030	0.35	−67.9	0.032	0.09	+83.3	0.017

**Table 13:** Results of the mechanical tests of the composites.

Sample	Husk	Slump			Specific Gravity			Rc, 28 Days			Rf, 28 Days		
Units	%	mm	C.D (%)	S.D	g/cm^3^	C.D (%)	S.D	MPa	C.D (%)	S.D	MPa	C.D (%)	S.D
Standard		137		0.202									
45_0_0	0	106	−22.1	1.291	2.23		0.001	51.92		2.224	7.93		0.898
45_0_12	0	114	−16.7	3.504	2.24	+0.3	0.005	57.46	+10.7	2.837	9.06	+14.2	0.735
45_0_20	0	109	−20.0	0.681	2.25	+0.3	0.004	53.02	+2.1	2.129	7.36	−7.2	0.419
45_50_0	50	116	−15.4	2.482	1.77	+0.6	0.006	9.30	−82.1	0.926	3.49	−56.0	0.184
45_50_12	50	115	−16.0	2.654	1.75	−20.8	0.108	17.50	−66.3	1.012	5.05	−36.4	0.159
45_50_20	50	128	−6.6	3.985	1.76	−21.4	0.113	17.21	−66.9	1.145	5.21	−34.4	0.456
45_100_0	100	87	−36.3	5.394	1.69	−21.2	0.026	0.33	−99.4	0.032	0.21	−97.4	0.022
45_100_12	100	85	−37.7	2.351	1.50	−24.1	0.010	1.64	−96.8	0.183	0.79	−90.1	0.158
45_100_20	100	82	−40.1	1.611	1.46	−34.8	0.018	2.33	−95.5	0.351	0.93	−88.3	0.079
50_0_0	0	131	−4.1	2.621	2.19		0.007	48.59		2.720	10.11		0.717
50_0_12	0	149	+9.1	5.650	2.21	+0.9	0.004	52.59	+8.2	2.550	11.09	+9.7	1.259
50_0_20	0	173	+26.9	2.804	2.22	+0.9	0.003	48.77	+0.4	2.265	10.15	+0.4	1.089
50_50_0	50	144	+5.3	1.966	1.71	+1.4	0.003	9.96	−79.5	0.705	3.67	−63.7	0.464
50_50_12	50	144	+5.5	6.058	1.80	−21.9	0.026	16.40	−66.2	0.515	4.38	−56.7	0.993
50_50_20	50	150	+9.7	3.790	1.74	−17.8	0.034	14.08	−71.0	0.666	4.69	−53.6	0.073
50_100_0	100	106	−22.1	1.291	1.51	−20.8	0.013	0.35	−99.3	0.051	0.26	−97.4	0.068
50_100_12	100	112	−17.8	1.208	1.30	−31.0	0.023	3.16	−93.5	0.441	1.67	−83.5	0.308
50_100_20	100	109	−20.0	0.681	1.31	−40.1	0.010	4.08	−91.6	0.740	2.06	−79.6	0.392
60_0_0	0	227	+66.2	1.888	2.19		0.008	45.77		2.048	8.41		0.839
60_0_12	0	231	+68.8	3.797	2.21	+0.6	0.003	47.38	+3.5	1.027	9.09	+8.1	0.499
60_0_20	0	236	+73.0	2.837	2.17	+0.6	0.085	43.71	−4.5	1.536	7.77	−7.6	0.580
60_50_0	50	188	+37.9	0.748	1.63	−1.0	0.005	4.92	−89.2	0.241	1.61	−80.9	0.269
60_50_12	50	201	+46.9	3.831	1.61	−25.6	0.002	7.33	−84.0	0.357	2.47	−70.6	0.473
60_50_20	50	204	+49.2	3.350	1.63	−26.4	0.022	7.72	−83.1	0.816	2.80	−66.7	0.245
60_100_0	100	88	−35.5	0.448	1.67	−25.7	0.046	0.59	−98.7	0.086	0.57	−93.2	0.072
60_100_12	100	91	−33.2	3.469	1.50	−24.1	0.009	1.64	−96.4	0.139	0.99	−88.2	0.313
60_100_20	100	92	−32.8	2.230	1.48	−32.7	0.017	2.43	−94.7	0.516	1.08	−87.2	0.359

## Data Availability

Data are available upon request due to restrictions of privacy.
